# Delivery of a Muscle-Targeted Adeno-Associated Vector Via *Ex Vivo* Normothermic Perfusion Is Efficient, Durable, and Safe in a Preclinical Porcine Heart Transplant Model

**DOI:** 10.3389/ti.2025.13971

**Published:** 2025-06-02

**Authors:** Krish C. Dewan, Jeng-Wei Chen, Alejandro A. Lobo, Ryan T. Gross, Chunbo Wang, Karla G. Rivera, Keely Dieplin Tran, Smith Ngeve, Violet G. Johnston, David Wendell, Carolyn K. Glass, Amy Evans, Sam Ho, Paul Lezberg, Widler Casy, Marla Bazile, Kruti Patel, Adam S. Cockrell, Carmelo A. Milano, Dawn Bowles

**Affiliations:** ^1^ Department of Surgery, Duke University Medical Center, Durham, NC, United States; ^2^ Department of Surgery, National Taiwan University Hospital, Taipei, Taiwan; ^3^ Duke Cardiovascular Magnetic Resonance Center, Duke University Medical Center, Durham, NC, United States; ^4^ Department of Pathology, Duke University Medical Center, Durham, NC, United States; ^5^ Perfusion Services, Duke University Medical Center, Durham, NC, United States; ^6^ Gift of Hope Organ and Tissue Donor Network, Itasca, IL, United States; ^7^ TransMedics, Inc., Andover, MA, United States; ^8^ Solid Biosciences, Charlestown, MA, United States

**Keywords:** adeno-associated virus vector, gene therapy, heart transplantation, *ex vivo* heart preservation, transgene durability

## Abstract

Normothermic *ex-vivo* organ perfusion (EVP) systems not only provide a physiological environment that preserves donor organ function outside the body but may also serve as platforms for *ex-vivo* organ modification via gene therapy. In this study, we demonstrated that a rationally designed muscle-tropic recombinant AAV, AAV-SLB101, delivered to the donor heart during brief normothermic EVP achieves durable cardiac transgene expression out to 90 and 120 days post-transplant in a porcine preclinical model. Moreover, transgene expression was detectable as early as 48 h post-transplant. Histological and MRI analyses of the donor myocardium showed no functional or structural impact on the allograft and no off-target gene expression in the recipient. This work will serve as a critical foundation to inform translational studies with therapeutic transgenes to improve allo-, xeno-, and auto-heart transplant outcomes.

## Graphical Abstract

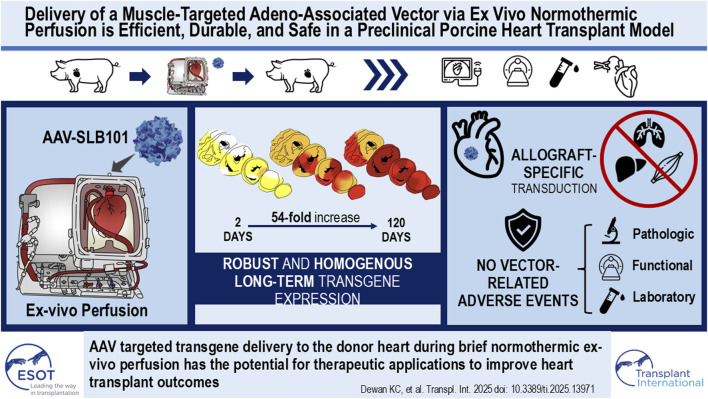

## Introduction

Heart transplantation (HT) remains the gold standard treatment for patients with advanced heart failure with a median overall survival that exceeds 12 years [1]. Early ischemia-reperfusion injury, primary graft dysfunction, chronic rejection, and debilitating side effects from long-term general immunosuppression remain key limitations. Gene therapy provided to a donor organ *ex-vivo* may provide a promising avenue to address these limitations and improve outcomes after HT.

Normothermic *ex-vivo* organ perfusion systems, such as the Organ Care System (OCS™; TransMedics, Inc., Andover, MA), create a physiological environment that perfuses and preserves donor organ function outside the body. While these systems are currently used to decrease ischemic time and facilitate greater organ utilization from longer distances, *ex-vivo* perfusion (EVP) may also serve as a platform for organ modification via advanced therapeutics such as gene therapy [2–8]. The applications of such an approach are numerous, including: i) countering rejection or ischemic-reperfusion injury ii) genetic modification of allogeneic donor hearts to expand the donor pool; iii) genetic modification of pig donor hearts to facilitate xenotransplantation; or iv), autotransplantation after EVP correction of genetic predispositions for advanced heart failure. Importantly, EVP creates an isolated environment for genetic modification of the donor heart, which avoids potential biological complications that could arise in a recipient host from unwanted biodistribution of a heart-specific genetic therapeutic administered systemically.

Adeno-associated viral (AAV) vectors have emerged as the preferred delivery modality for many currently approved gene therapies due to their favorable safety profile, tissue-specific tropism, durable efficacy, and manufacturability [9]. In our previous work using a pig heterotopic HT model, we successfully delivered the firefly luciferase transgene to donor hearts through EVP with recombinant AAV vectors (SASTG, a myocardial-enhanced AAV3b variant) [8]. At 30-day follow-up, we demonstrated consistent, dose-dependent protein expression and vector DNA in the allograft without off-target effects [8]. It remains unclear how soon after transplantation transgene expression begins and how long transgene expression persists in the allograft.

In this study, we demonstrate robust, early-onset, and durable gene expression from 2 to 120 days after transplantation following delivery of a novel muscle-specific recombinant AAV capsid, AAV-SLB101, in our laboratory’s preclinical porcine heterotopic heart transplant model [8, 10–13].

## Materials and Methods

### Animals and Vector

The proprietary AAV-SLB101 capsid, containing a vector expressing firefly luciferase from the CK8 promoter, was obtained from Solid Biosciences Inc., (Charlestown, MA). Vector production is described in the Supplementary Material. Female Yucatan pigs (Sinclair Bio Resources, Auxvasse, MO), aged 6–8 months and weighing 20–40 kg, were selected based on matched swine leukocyte antigen (SLA) Class I and Class II genotyping and compatible blood typing. Four pairs of pigs were utilized in this study. This study was approved by the Duke University Institutional Animal Care and Use Committee.

### Model of Heterotopic Heart Transplantation and Vector Delivery

The porcine heterotopic HT model has been previously described [8]. The AAV dose used for each donor heart was 1 × 10^14^ viral genome copies (VGCs). The donor heart was subjected to 2 h of EVP during which perfusate was collected at regular intervals. After EVP, the donor heart was arrested using Del Nido cardioplegia and implanted into the recipient pig’s abdomen, with aorto-aortic and pulmonary artery-to-inferior vena cava anastomoses as previously described [8].

Recipient pigs were treated with maintenance immunosuppression, including daily methylprednisolone for 14 days (rapidly tapered from 24 mg/kg twice daily on postoperative day 1 to 1 mg/kg once daily by day 14), daily mycophenolate mofetil 500 mg for 14 days and daily intramuscular injected tacrolimus (0.1 mg/kg to begin titrated from there onward to maintain therapeutic serum trough levels) until termination.

### Graft Evaluation

Allograft function was examined twice daily by physical palpation and rated on a four-point subjective scale and point-of-care ultrasound. Both donors and recipients underwent preoperative (3 days prior to surgery) and postoperative (day 12) cardiac MRI (CMR) to assess function, fibrosis, and edema. This was performed via T1 mapping, DE-CMR, and assessment of allograft size and function as described previously [14]. A transcatheter endomyocardial biopsy was performed at 30 and 60 days postoperatively [15]. Blood was drawn at biweekly timepoints for tacrolimus trough levels, laboratory panels (comprehensive metabolic panel, complete blood count with differential, and troponin), and for plasma for future analyses. Plasma was obtained by centrifugation of whole blood at 11,000 rpm and isolation of the supernatant fraction. This was flash frozen in liquid nitrogen and stored at −80°C.

### Euthanasia and Terminal Cardiac Tissue Preparation

Under sedation and mechanical ventilation, the allograft and native heart individually received 500 mL of Del Nido cardioplegia and were rapidly explanted into ice cold PBS. Various parts of the allograft and native recipient heart were collected. Each heart was sectioned into five breadloaf-like cross sections labeled 1–5 from base to apex respectively. Section *Introduction* represented both atria. The atria were sampled from three different portions: right atrium (RA), left atrium (LA), and interatrial septum (SA). The ventricular portion was horizontally sliced into four sections from the atrioventricular groove to the heart apex, labeled as ventricular cross sections 2, 3, 4, and 5 (Apex). Ventricular cross sections 2, 3, and 4 were further divided into three portions: anterior wall, middle wall, and posterior wall and were sampled from left ventricle (LV), right ventricle (RV) and interventricular septum (IVS). In total, 31 myocardium tissue samples from different sections were collected for each heart.

### Tissue and Plasma Analysis

Transgene expression and vector transduction of tissue samples from the allograft, native heart, and extracardiac organs were assessed using luciferase activity assays, immunofluorescence staining, and DNA isolation/analysis for vector copy number. Lastly, anti-vector antibody assessment was performed using an ELISA assay. Detailed protocol descriptions of can be found in the Supplementary Material.

### Statistical Analysis

For descriptive statistics, means and standard deviations were calculated. Comparisons were performed using either Mann–Whitney U, Fisher’s exact, and chi-square tests where appropriate. For comparison of continuous variables, Student’s t-test or Mann-Whitney U test were used for parametric or non-parametric data respectively. A one-way ANOVA was used for multiple comparisons using Tukey’s post-hoc tests. All statistical analyses and graph creation were performed using GraphPad Prism version 10.2.3 (GraphPad Software, San Diego, CA). Statistical significance was defined as P < 0.05.

## Results

### Heterotopic Pig Heart Transplant Perioperative Conditions and Outcomes

**FIGURE 1:**
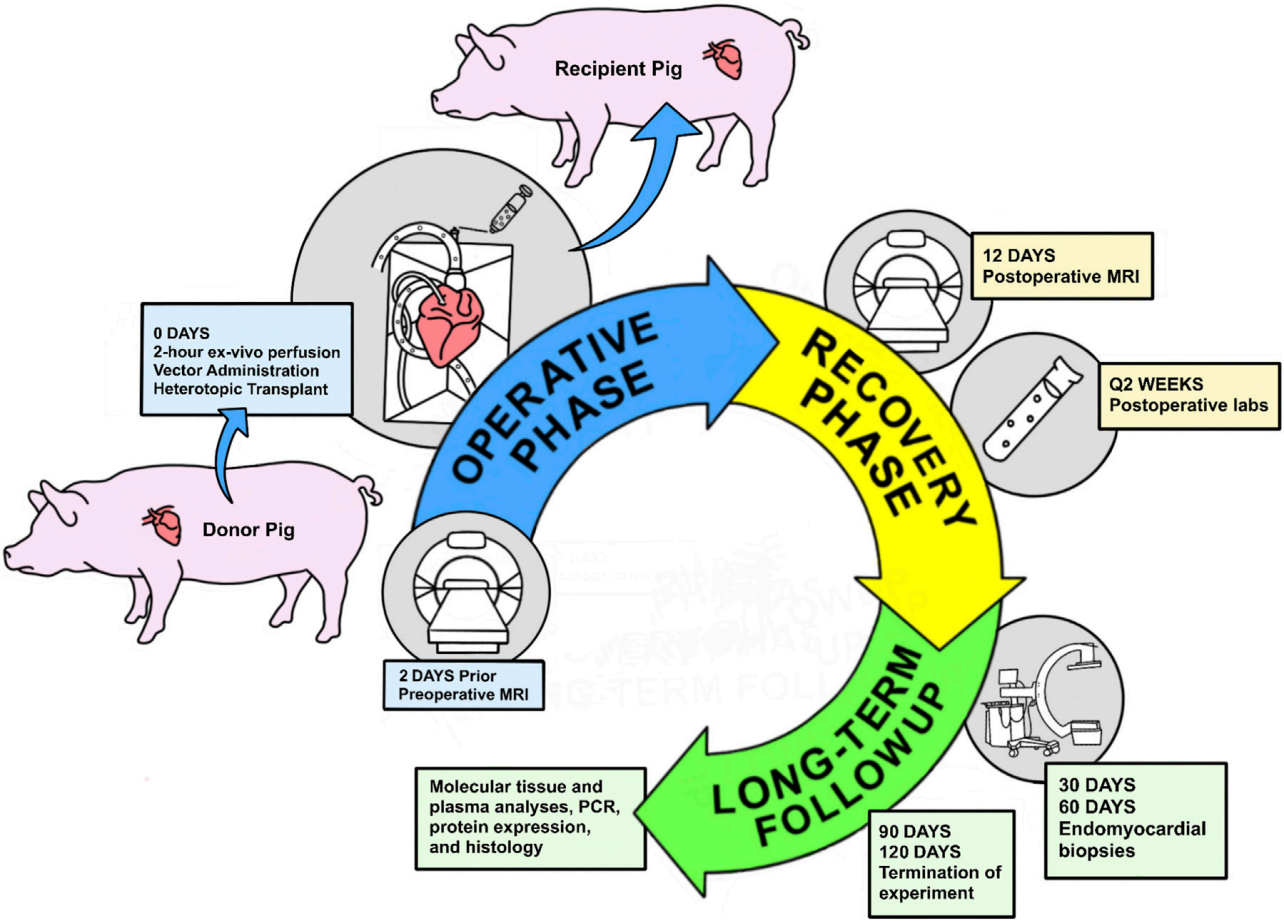
Overview study design of animal experiments and analysis pipeline.

The overall study design is summarized in Figure 1. Four pigs underwent heterotopic HT. Two pigs encountered perioperative complications (delayed bleeding and primary graft dysfunction) requiring early termination of the experiment at 48 and 24 h respectively. The remaining two pigs survived until the designated study endpoints, with follow-up durations of 90 and 120 days, respectively. The general appearance and activity levels of these two long-term survival pigs were unremarkable throughout the study period. Routine laboratory tests, including complete blood count (CBC) and comprehensive metabolic panel (CMP) remained within normal limits overall (Supplementary Figures S1, S2). Troponin was only appropriately elevated in the immediate postoperative period due to surgical manipulation but normalized for the remainder of the study (Supplementary Figure S7). Minor elevations in in blood urine nitrogen and creatinine in one recipient normalized after reduction of tacrolimus (Supplementary Figure S2). There was no evidence of renal impairment or change in urinary output.

### Early Peak and Subsequent Decline of Vector DNA in Cardiac Tissue Post-Transplant

**FIGURE 2:**
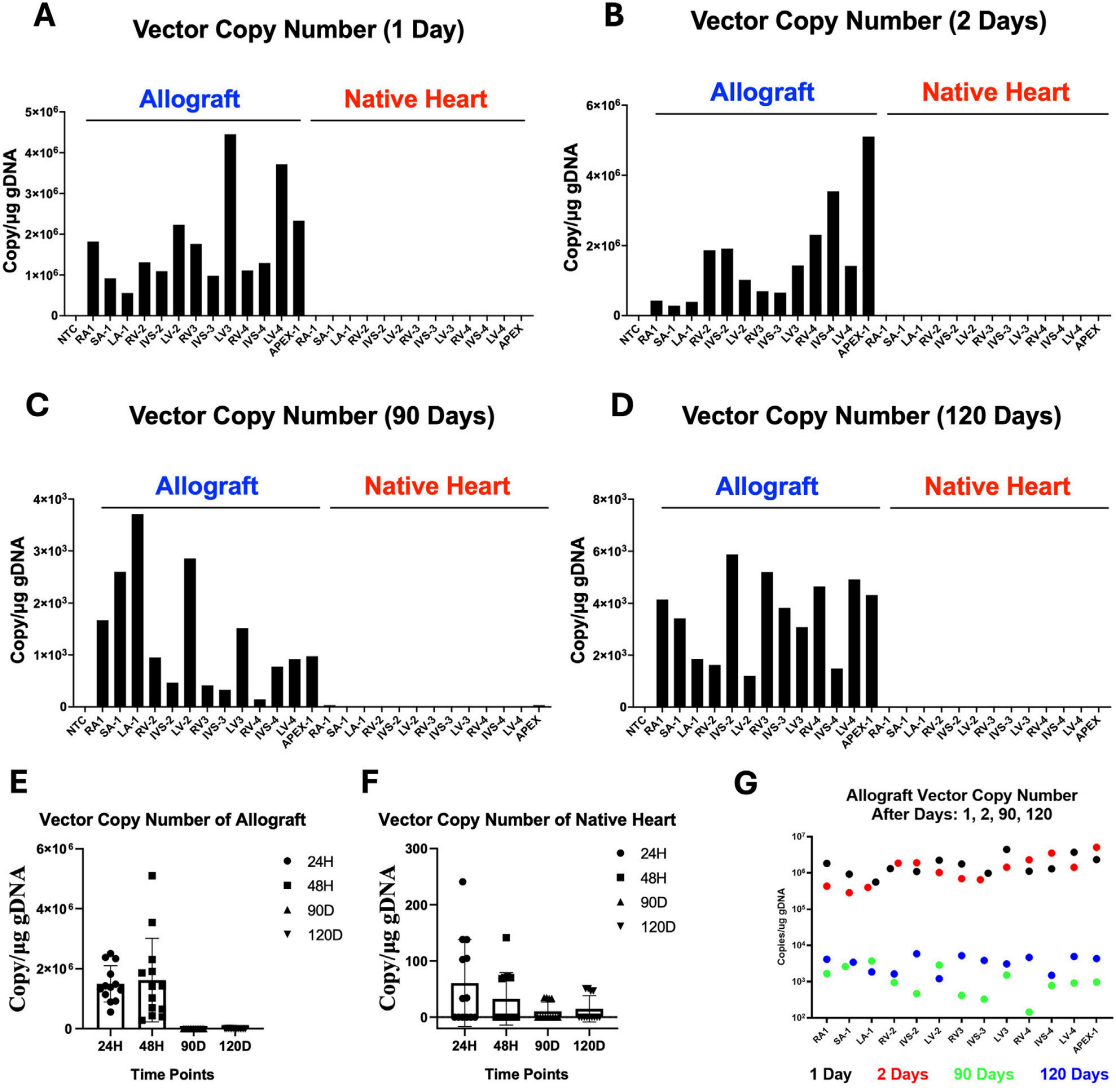
Vector DNA over time. Viral genome copy numbers per µg DNA in each chamber of the transplanted and native hearts at various timepoints after transplantation **(A)** 1 day **(B)** 2 days **(C)** 90 days, and **(D)** 120 days. Samples are labeled along with the number of the cross section from which they were obtained. Cross sections are numbered 1–5 from base/atrium of the heart to apex respectively. All samples were obtained from the medial aspect of each chamber for consistency. **(E)** Average vector copy number across the entire allograft at 24 h, 48 h, 90 days and 120 days. **(F)** Negligible viral DNA at any timepoint in any of the native untreated hearts. **(G)** Side-by-side comparison of vector DNA copy numbers by chamber over time. LA, left atrium; SA, atrial septum; RA, right atrium; LV, left ventricle; RV, right ventricle; IVS, interventricular septum; NTC, no template control.

The presence of vector DNA in cardiac and extra-cardiac tissues was measured in all pigs using qPCR. To compare the viral genome particles in heart tissues, the atrium, anterior portion of the ventricle, septum, and apex were analyzed (Figures 2A–D). In the allograft, the viral genome DNA copies significantly decreased over time (Figure 2E, P < 0.001 by ANOVA, Figure 2G). In the native heart, the average viral genome DNA copies were negligible across different time points (Figure 2F, P = 0.0382 by ANOVA). For extra-cardiac organs, most samples had no detectable viral DNA. Only the liver, lung and psoas muscle contained low amounts at the early time points (1512 VGC/μg in the lung and 447 VGC/μg in the liver at 24 h, 70 VGC/μg in liver, and 69 VGC/μg in psoas muscle at 48 h). No viral DNA was detected in extra-cardiac organs at 90 and 120 days.

### No Change in Vector DNA in the Perfusate Over Time During *Ex-Vivo* Perfusion

The presence of vector DNA in perfusate from the TransMedics system for all four animals was measured using qPCR. Across all timepoints from 0 to 90 min, there was no significant difference in vector copy numbers (p = 0.06; Supplementary Figure S8). A detailed characterization of relevant lab, drug, and timing parameters during the 2 h EVP period are provided in Supplementary Table S1.

### Homogenous Durable Transgene Expression and Activity Observed Post-Transplant

Tissue samples from 31 areas of the allograft, including the LA, RA, SA, LV, RV, IVS, and apex, were analyzed for luciferase activity using a luminometerComplete data sets of individual luciferase activity measurements at different time points and regions of the heart are provided in Supplementary Table S2.

**FIGURE 3:**
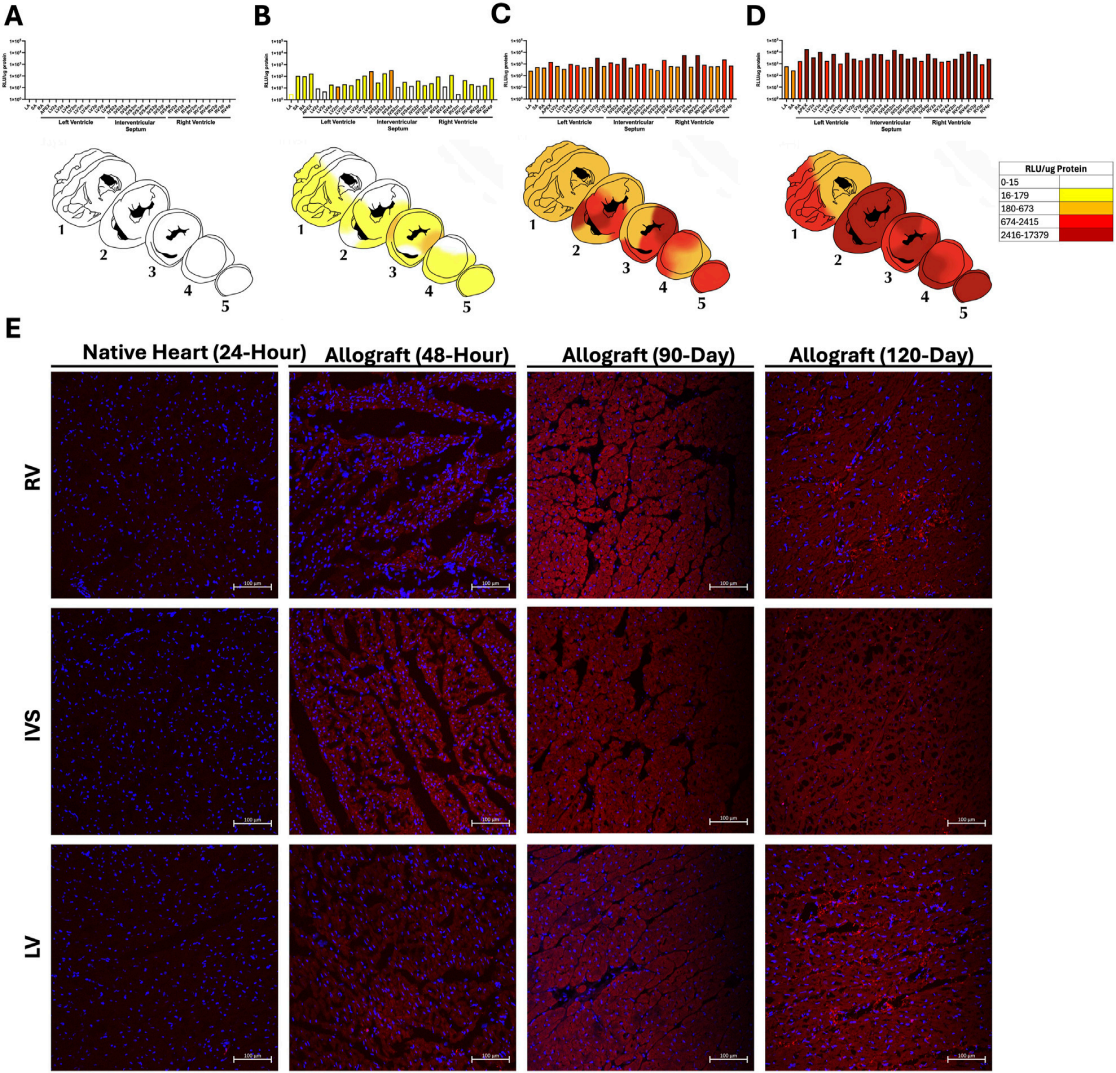
Luciferase expression throughout the myocardium. Luciferase expression at **(A)** 1 day **(B)** 2 days **(C)** 90 days and **(D)** 120 days expressed as relative light units (RLU) per ug tissue protein. Transgene expression is displayed both on the graphs numerically as well as in the heatmap. Graphs are plotted on the log10 scale. **(E)** Representative histology with immunofluorescent staining of luciferase(red) and counter staining the nucleus with DAPI (blue). All images are of medial wall from the third cross-section at ×20 magnification, with the scale bar indicating 100 μm. LA, left atrium; SA, atrial septum; RA, right atrium; LV, left ventricle; RV, right ventricle; IVS, interventricular septum. Nomenclature of chamber: chamber, cross section, wall (e.g., LV2a was a left ventricular sample from the anterior aspect of cross section 2).

In the allograft of the 24-h pig, no significant luminescence signal was detected in any of the chambers (Figure 3A; Supplementary Table S2). However, in the 48-h pig, enzyme activity was detectable throughout the allograft (Figure 3B; Supplementary Table S2). Immunofluorescence staining of the allograft confirmed the expression of luciferase throughout the allograft, in contrast to the native recipient’s heart, which showed no signal on the luciferase assay (Figures 3C–E; Supplementary Figure S4). Allografts examined at 90 days and 120 days post-transplant showed even more robust luciferase activity and immunofluorescence staining (Figure 3E; Supplementary Figures S4, S5).

**FIGURE 4:**
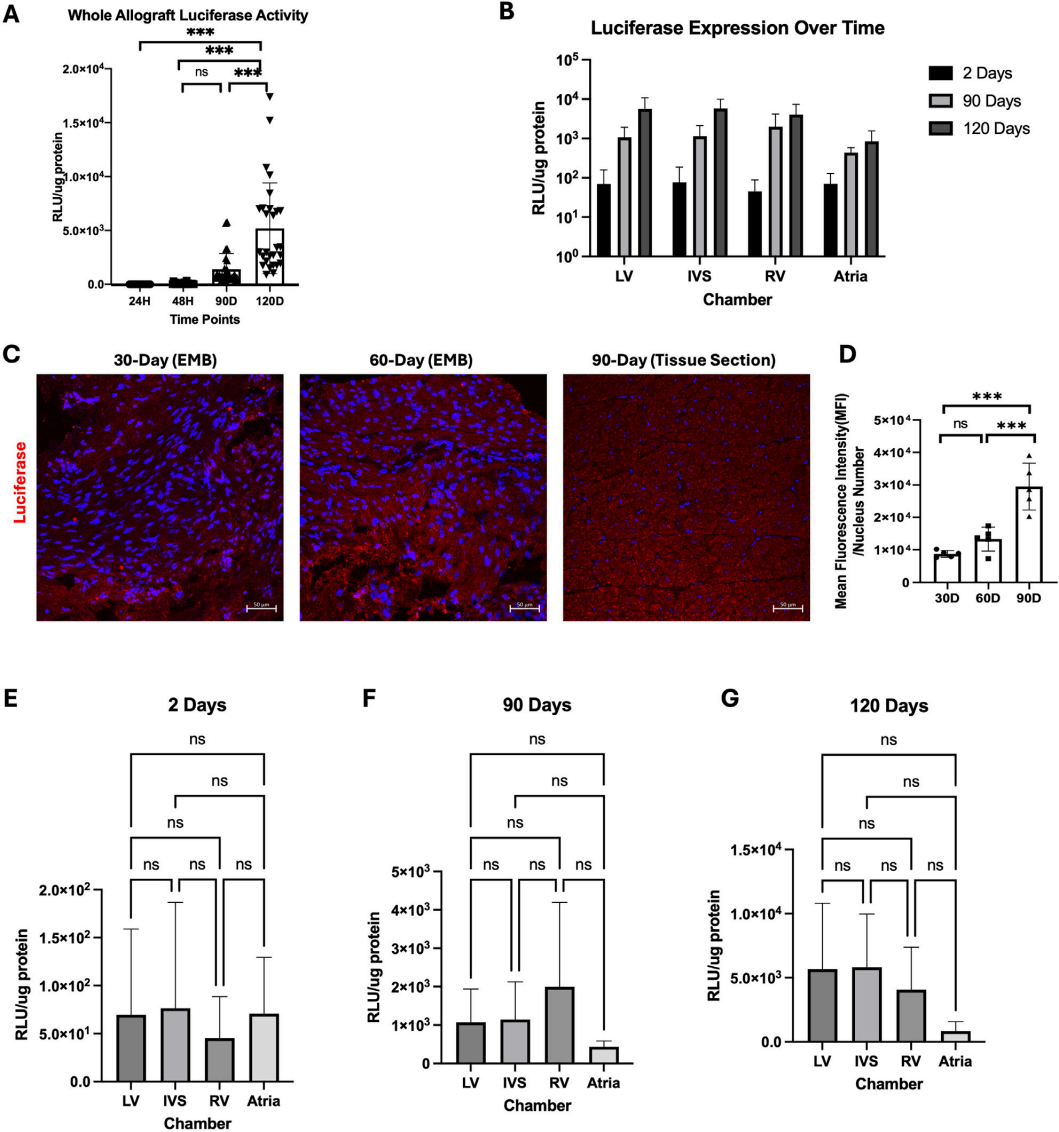
Luciferase biodistribution and progression of expression over time. **(A)** Overall luciferase expression from 1 to 120 days assessed by luciferase activity assay. **(B)** Luciferase expression over time by chamber. Graphs are plotted on the log10 scale. **(C)** Representative luciferase staining of endomyocardial biopsies. **(D)** Mean fluorescent intensity (MFI) per nucleus of luciferase among endomyocardial biopsy samples at 30, 60, and 90 days. Luciferase enzymatic activity across all chambers (atria and ventricles) at all three timepoints from 2 days **(E)** to 90 days **(F)** to 120 days **(G)** post-transplant. RLU values were calculated by pooling values for all samples from each chamber (n = 3 atria, n = 10 LV, n = 9 IVS, n = 9, IVS). LV, left ventricle; IVS, interventricular septum; RV, right ventricle.

Luciferase activity increased significantly over time, with the luminescence intensity being much higher in the 120-day allograft compared to the 24-h, 48-h, and 90-day allografts (P < 0.001 by ANOVA; Figure 4A). Luciferase activity increased 54.4-fold in the LV, 334.8-fold in the IVS, 46.5-fold in the RV, and by 11-fold increase in the atria from 2 to 120 days (Figure 4B). The same was true of tissues analyzed from the same allograft via endomyocardial biopsies (EMBs) at 30 days and 60 days (septal wall of the RV) along with final collected sections after euthanasia at 90 days (RV wall). The signal intensity at 30 days was 136-fold stronger than that seen at 48 h (p < 0.001; Figures 4C,D). Signal intensity was significantly increased at 90 days compared to both 30-day or 60-day timepoints (P < 0.001 by ANOVA). There was no statistically significant difference in signal intensity at 60 days compared to 30 days (P = 0.3124 by ANOVA) (Figure 4D).

The biodistribution of luciferase activity across the allograft was also equal with no significant differences between chambers of the heart at 48 h (P = 0.215, Figure 4E). 90 days (P = 0.73) or 120 days (P = 0.10) (Figures 4F,G).

### Absence of Off-Target Protein Expression and Demonstrated Safety of Vector Delivery

**FIGURE 5:**
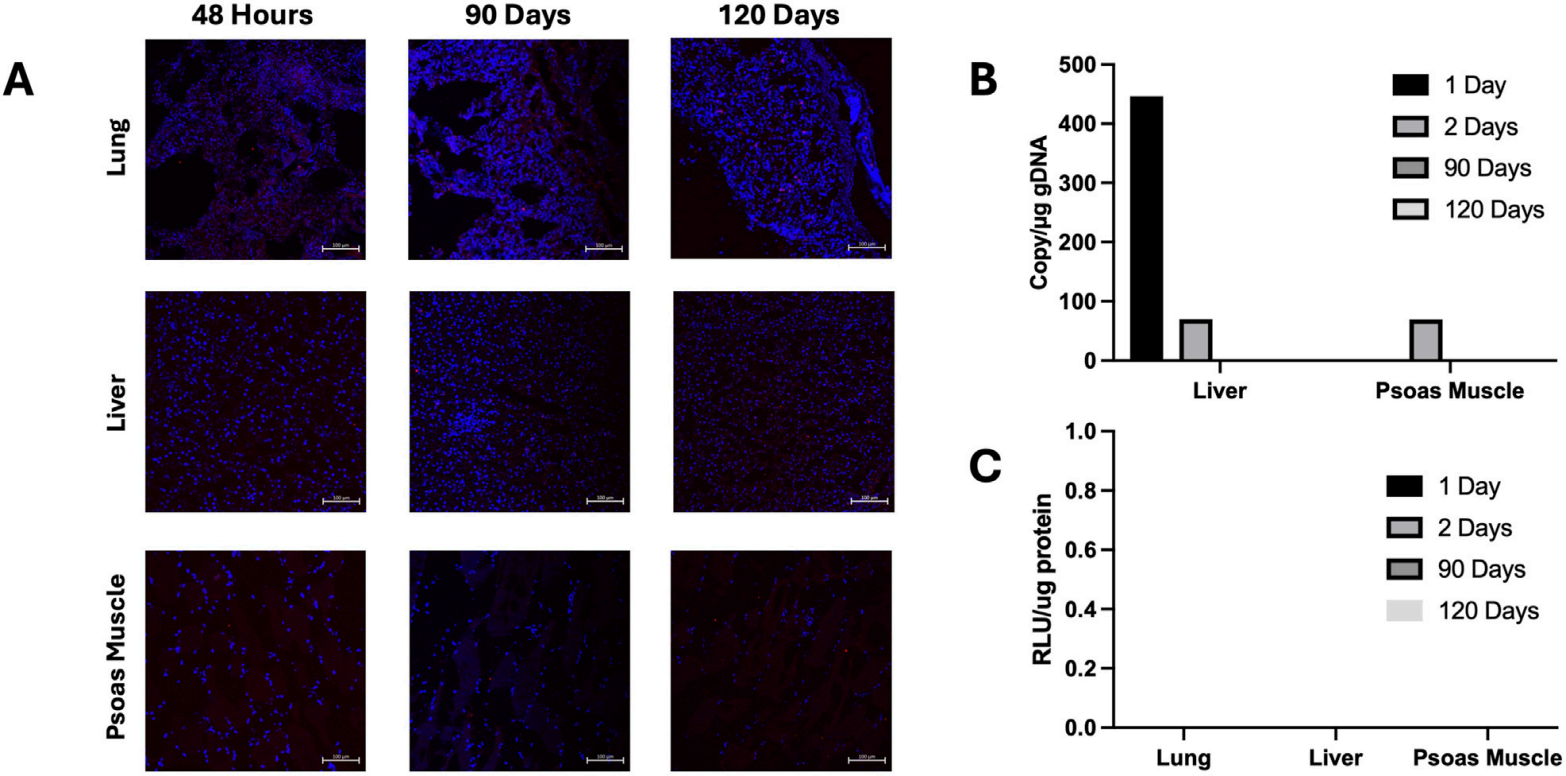
Luciferase expression in extra-cardiac organs. **(A)** Luciferase immunofluorescent staining of recipient extra-cardiac organs including the liver, lung, or skeletal muscle at any timepoint after transplantation. All images are at ×20 magnification, with the scale bar indicating 100 μm. Psoas m. = psoas muscle (representative of skeletal muscle). **(B)** Viral copy numbers per ug DNA in the liver and psoas muscle at 24 h, 48 h, 90 days, and 120 days **(C)** Luciferase activity at all timepoints in the lung, liver, and skeletal muscle.

The recipient’s extra-cardiac organs, including the lung, liver, kidney, and psoas muscle, were analyzed for off-target gene expression. The psoas muscle was included due to its proximity to the allograft in this heterotopic model. Although vector DNA was detected in the liver and psoas muscle of the recipients from the 24-h and 48-h survival studies (Figure 5B), both the luciferase enzymatic assay and immunofluorescence staining demonstrated no protein activity or expression in any of the study pigs (Figures 5A,C).

**FIGURE 6:**
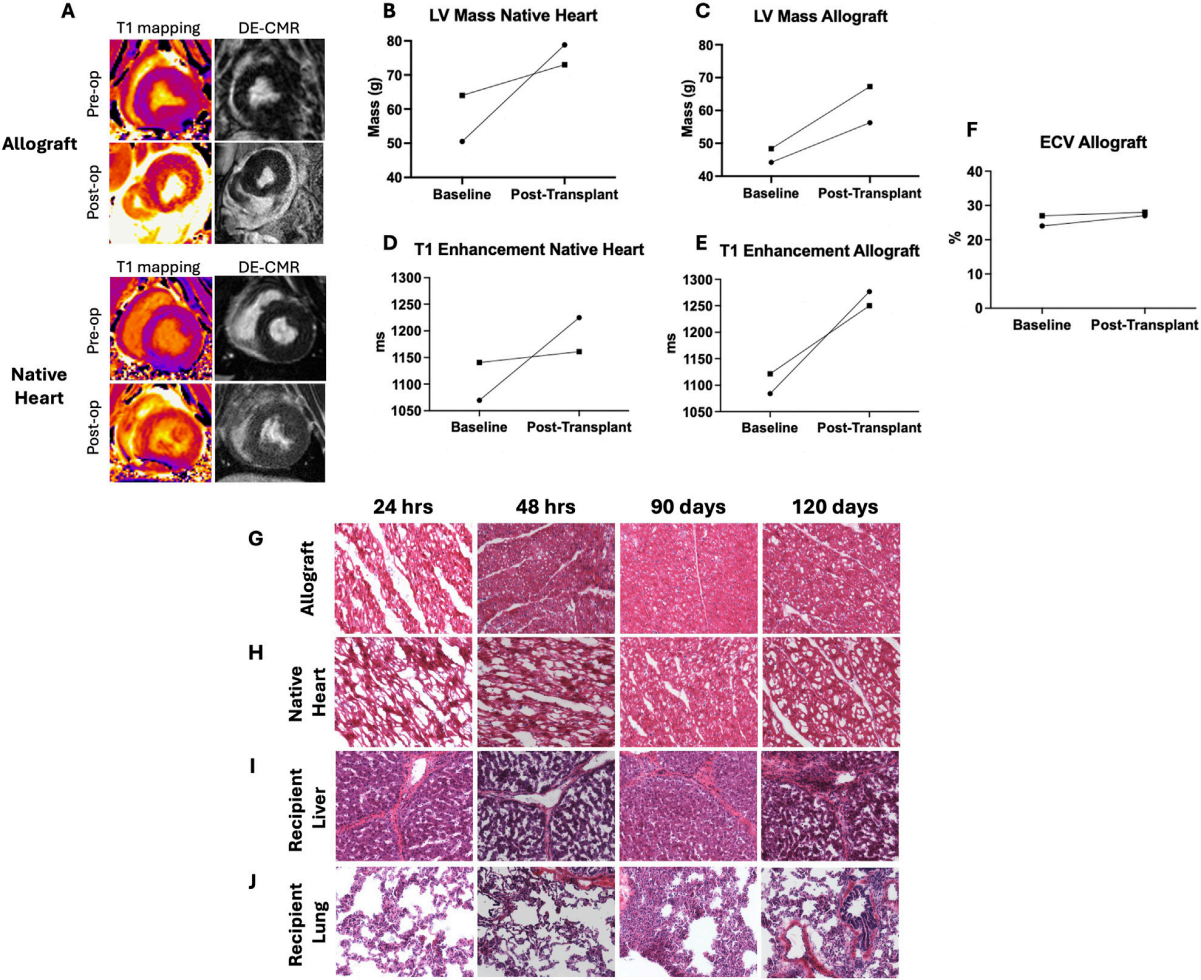
Safety of ex vivo vector delivery via cardiac MRI and histopathologic assessment. **(A)** Representative preoperative and postoperative (day 12) CMR T1 mapping and DE-CMR images. **(B)** Change in left ventricular mass in the native heart **(C)** Change in left ventricular mass in the allograft **(C)**. **(D)** Changes in T1 enhancement in the recipient native heart **(E)** Changes in T1 enhancement in the allograft. **(F)** Extracellular volume (ECV) in the allograft. H&E staining of the recipient’s allograft **(G)** native heart **(H)**, liver **(I)**, and lung tissue **(J)**.

To assess the impact of AAV-SLB-101-mediated gene delivery on allograft function, echocardiography, cardiac MRI, and histological analysis of myocardium were performed on the pigs survived to 90 and 120 days respectively. Echocardiography consistently demonstrated robust allograft contractility throughout the study period. Pre- and post-operative cardiac MRI revealed no signs of fibrosis on delayed-enhancement cardiac magnetic resonance imaging (DE-CMR) scans and no tissue edema on T1 mapping (Figures 6A–E). Additionally, the extracellular volume (ECV) values remained stable before and after the operation (Figure 6F). LV mass was calculated by a formula that takes into account myocardial volume, thickness, and density. Increase in native heart LV mass was commensurate with the growth of the animal itself. Increases in heterotopic allograft mass were more reflective of expected underfilling and resulting “pseudohypertrophy” of the LV rather than a true LV mass increase. Histological examination with H&E staining of the allograft and the recipient organs demonstrated no overt signs of myocarditis, inflammation, rejection, or compromise in structural integrity as reviewed by a board-certified cardiac pathologist (Figures 6G–J; Supplementary Table S4). Additionally, there was neither elevation in cardiac troponin in any recipient, outside of the immediate postoperative period, nor elevation of functional liver laboratory values throughout the study period (Supplementary Figure S7).

### Anti-AAV-SLB101 Capsid Responses Peaked Early and Declined Over Time

The minimally detectable levels of vector DNA in extra-cardiac tissues indicated that minor amounts of vector may leach from the transplanted donor heart into the recipient circulation, which may potentially elicit an antibody response to AAV-SLB101 capsid proteins. The titer of circulating anti-AAV-SLB101 capsid antibodies in plasma of recipient was measured utilizing ELISA (Supplementary Figure S9). There was a 3-fold increase in anti-AAV-SLB101 capsid antibodies in both recipients from baseline to 12 days. For one of the two long-term pigs, the titer declined 46-fold by 120 days to a level comparable to baseline while the other’s titer decreased 1.5 fold from peak but remained higher than baseline.

## Discussion

This study demonstrated that the muscle-tropic AAV, AAV-SLB101, delivered to the donor heart during a short 2-h normothermic EVP can be effective for achieving early-onset, durable, and targeted cardiac transgene expression without functional or structural impact on the allograft and off-target gene expression. Currently, the Food and Drug Administration (FDA) has approved a total of 14 *ex-vivo* and 29 *in-vivo* gene therapies as safe and effective treatments for genetic disorders [9]. Of these, 89% utilize viral vectors including adenovirus, AAV, herpes simplex virus, γ-retroviral and lentiviral vectors [16]. Among these, AAV vectors have emerged largely as the vector of choice in gene therapies due to their favorable safety profile, durable therapeutic delivery, and precedence for clinical regulatory paths [17]. While a lentiviral vector may allow for longer gene expression to target rapidly-dividing cells (e.g., endothelial cells) or may accommodate larger genetic cargo, cardiomyocytes have very low turnover rates. In addition our proposed therapeutic targets are small enough to be accommodated by an AAV. Therefore, an AAV vector was selected for this study.

There is strong potential for EVP as a platform for gene therapy during organ transplantation [4–11]. *Ex-vivo* administration is attractive in the setting of solid organ transplantation for a number of reasons that we have demonstrated in this preclinical swine model, including: i) quick onset of expression following brief perfusion; ii) homogeneity of transduction throughout the heart; iii) durability of expression; iv) and mitigation of vector-related adverse events in the recipient due to a near absence of vector load in extra-cardiac tissues. Similar approaches utilizing EVP in preclinical models have been applied to lung, liver, and kidney transplantation [18–26]. Together, these studies support the use of AAV vectors to deliver therapeutics for *ex vivo* donor organ modification before transplant into the recipient with applications including decreasing allograft immunogenicity to mitigate use of general immunosuppressants, prevent ischemia-reperfusion injury, expanding the allogeneic donor pool, and optimizing xenografts for improved transplant outcomes.

AAV-SLB101 was designed with the goal of preferentially targeting and transducing striated muscle cells following systemic administration [12, 13]. In preclinical animal studies, AAV-SLB101 demonstrated effective transduction and biodistribution to the skeletal muscle and heart and is currently under investigation in a clinical trial for Duchenne muscular dystrophy [12, 13]. We demonstrate, for the first-time, transduction and biodistribution of AAV-SLB101 throughout the swine heart with *ex vivo* perfusion. We demonstrate that transduction during EVP can restrict tropism to the allograft donor heart, and not the representative skeletal muscle (psoas) or native heart from the recipient pig. Luciferase was expressed throughout the transplanted myocardium at all timepoints in all chambers from as early as 2 days to as late as 120 days post-transplant.

Neither the transplanted heart nor recipient organs were subject to vector-related inflammation or myocardial compromise as examined by pathology and CMR assessment. CMR-based myocardial tissue characterization has recently emerged as a non-invasive method to clinically assess transplanted cardiac allografts in human patients for rejection, inflammation, myocarditis, and fibrosis. In particular, T1 and T2 mapping correlate well with histopathological evaluation and are highly sensitive for detecting fibrosis and edema [27]. Allograft edema (as measured by T1 enhancement) and fibrosis (measured by via delayed-enhancement CMR, DE-CMR) increased comparably between the native heart and the transplanted allograft due to post-surgical changes not related to acute rejection. Indeed, a similar phenomenon is observed clinically [28]. The ECV of the allograft both before and after vector exposure/transplantation remained within the standard expected value of 25.3 ± 3.5%, once again indicating a lack of vector-mediated fibrosis or inflammation [29]. Functional cardiac assessment in this study was limited by the fact that these allografts were transplanted heterotopically. Hence in an unloaded state, the change in ejection fraction or other typical functional assessments are not reliable. However, ECV and T2 mapping correlated with functional measurements such as systolic longitudinal function, peak myocardial velocities and desynchrony [28]. Given the correlation between structural and functional CMR measurements and the lack of changes in structural assessment (e.g., ECV) in our study, it is likely that vector delivery via EVP did not cause any measurable functional changes in the allograft.

Hepatotoxicity is a well-known adverse event attributed to AAV-mediated gene therapies as viral vectors pass through hepatic circulation when provided systemically [30, 31]. Hinderer et al described rapid asymptomatic transaminitis and bilirubin elevation after administration of 2 × 10^14^ VGC/kg of an AAV9 variant in 2 non-human primate (NHP) animals while one NHP exhibited profound transaminitis, systemic inflammation, disseminated intravascular coagulation (DIC), and shock leading to liver necrosis and death [32]. Similarly, Hordeaux et al. showed that at a dose of 7.5 × 10^13^ VGC/kg, mature NHPs developed acute liver enzyme elevation, evidence of DIC, and hemorrhage requiring euthanasia [33, 34]. In the current study, there were no elevations in liver enzymes throughout the study, nor evidence of end-organ damage aside from brief tacrolimus-associated nephrotoxicity despite a high dose of 1 × 10^14^ VGC. By avoiding exposure of the liver to AAV vector, EVP prevented the potential for hepatoxicity or other associated adverse events despite the high vector dose delivered to the transplanted donor heart. Though some viral DNA was seen in the liver at day one post-transplant, this was negligible compared to the allograft myocardium (liver 400 vs. > myocardium 4 × 10^6^ VCG at day one) and decayed rapidly over the next 24 h. Importantly, levels of vector detected in the liver are several thousand-fold lower than levels anticipated to result in hepatotoxic issues [30–34].

An analysis of viral DNA within the OCS perfusate over the entire period of perfusion demonstrated that the EVP circuit remained saturated with AAV throughout 90 min of EVP at the current dosage. The dosage of AAV used in this study, 1 × 10^14^ vg, was based on previous AAV3b experiments. Based on these findings, it may be possible to decrease the dosage of AAV-SLB101 while still achieving the same transduction and transgene expression. This adjustment could dramatically lower manufacturing costs and reduce the risk of AAV-associated immune responses.

We demonstrated persistent luciferase expression that increases over 120 days following heterotopic pig heart transplant. Though pigs were SLA-matched in this study, we utilized triple-regimen immunosuppression for a brief 2-week period to mitigate allograft rejection, the current standard of care for heart transplantation, followed by tacrolimus monotherapy [35]. Host innate and adaptive immune responses to the vector capsid and/or transgene have been reported in a number of preclinical and clinical studies which may negatively impact patient health, vector transduction efficiency, therapeutic durability and dosing strategies [36, 37]. These immune responses and resulting morbidity have led to the adoption of short-term systemic immunosuppressive regimens after AAV-gene therapy in clinical practice.

However, there is no standard immunosuppressive regimen for the suppression of anti-vector responses at present [29]. Corticosteroid regimens as short as 7 days to as long as 133 days have been used in 95% of clinical trials thus far, either alone or in combination with other immunosuppressive agents [38]. Tacrolimus and mycophenolate mofetil (MMF) were both used in 15% of studies. Tacrolimus and MMF are most helpful in mitigating the T-cell dependent acute cellular response that is often responsible for both reduced transgene expression and hepatotoxicity. The lack of detectable myocarditis in the donor hearts at 90 and 120 days support the conclusion that continued use of tacrolimus interfered with an anticipated inflammatory response to the foreign luciferase protein in the allograft.

The effect of pre-existing and/or *de novo* anti-vector antibodies remains unclear at this time. We found an initial increase in anti-vector antibodies postoperatively that subsequently decreased over time. This mirrors what has been seen in clinical trials where increases in anti-AAV antibodies were present regardless of AAV serotypes, administration route, and the presence of an immunosuppressant regimen [38]. The appropriate immunosuppressive regimen to target both cellular and humoral responses has yet to be defined. Despite this, our study demonstrates that transgene expression and overall safety of the recipient are unaffected by humoral responses even when present due to exogenous delivery of the vector to the allograft and a chronic single-agent immunosuppressive regimen.

While transgene expression remained robust and increased over time, the measurable vector DNA present within transduced allografts decreased over time. The inverse correlation between expression and vector DNA may be explained by cellular trafficking of AAV through the cytoplasm. Following cellular entry and transport of AAV particles through the endosomal/lysosomal pathway, vector particles may be either targeted for proteasomal degradation of the capsid in the cytoplasm or transported to the nucleus where capsid uncoating and release of the single-stranded DNA cargo occurs [39]. Ubiquitination of conserved tyrosine residues on the AAV capsid surface targets capsid particles for proteosome degradation, which may leave fewer capsids available to reach the nucleus [31]. Extrapolating proteosome degradation with concomitant nuclear entry of AAV-SLB101 over time supports our observation that the AAV-SLB101 vector DNA decreases over time as a consequence of proteosome degradation, while vector particles are also entering the nucleus and release the vector DNA payload resulting in increased luciferase expression. Hence it is possible that at the early post-EVP and transplant stage (i.e., day 1 and 2), much of the viral DNA measured is a combination of both cytoplasmic and nuclear vector genomic cargo while at the later time points (day 90 and 120) only the extrachromosomal nuclear DNA remains, thereby supporting increased luciferase expression.

There are still questions that remain unaddressed from this study that could serve as the foundation for future work. Assessment of vector-related markers of inflammation or endothelial activation in the allograft during EVP was not possible due to the confounder of donor-derived inflammatory markers in the perfusate. Using of non-donor derived blood for EVP perfusate in subsequent experiments will facilitate evaluation of this if present. The vector utilized in this study utilized a creatine kinase promoter. Hence, we would not expect it to show transgene expression in any other cells appreciably other than muscle. As a result, we were unable to assess cell-specific transduction within the allograft that are implicated in acute and chronic rejection such as the endothelium and fibroblasts. Our earliest timepoint was 24 h after transplantation. A baseline quantification of VCNs immediately after EVP may be obtained in future experiments by performing a core needle biopsy just prior to transplantation. Whether expression would persist after 120 days or whether there would be an immune response to the transgene in the absence of tacrolimus immunosuppression remains unclear. Removal of immune suppression would also provide a clear understanding of whether transgene expression would continue to increase or plateau and if AAV vector transduction was impacted. The high vector dose used in this study ensured transduction throughout the heart, but optimal effective dose was not determined. Reducing dose can result in enhanced safety and cost savings. Lastly, given the physiological changes associated with the unloaded state of the allograft, the use of a heterotopic model precludes the ability to assess functional changes to the heart. Although out of the scope of this manuscript development of a preclinical orthotopic model would be amenable to building a functional understanding of the allograft.

### Conclusion

Despite these limitations, this study demonstrates that utilizing a muscle-tropic AAV, AAV-SLB101, to modify the swine donor heart during normothermic *ex vivo* perfusion is effective, with transgene expression detectable as early as 48 h and persisting for at least 120 days without decay. Histological and MRI analyses showed no functional or structural impact on the allograft and no off-target gene expression in the major organs of the recipient. Future research should focus on refining immunosuppression protocols, exploring the long-term durability of gene expression beyond 120 days, and evaluation of dose-dependent long-term transgene expression. Overall, this approach holds significant potential for enhancing the outcomes of solid organ transplantation.

## Data Availability

The original contributions presented in the study are included in the article/Supplementary Material, further inquiries can be directed to the corresponding author.

## Abbreviations

HT, Heart Transplant; OCS, Organ Care System; EVP, *Ex Vivo* Perfusion; AAV, Adeno-Associated Virus; SLA, Swine Leukocyte Antigen; VGC, Viral genome Copies; CMR, Cardiac Magnetic Resonance Imaging; LA, Left Atrium; SA, Atrial Septum; RA, Right Atrium; LV, Left Ventricle; RV, Right Ventricle; IVS, Interventricular Septum; RLU, Relative Light Units; OCT, Optimal Cutting Temperature compound (OCT); ddPCR, droplet polymerase chain reaction; CBC, complete blood count; CMP, comprehensive metabolic panel; ANOVA, Analysis of Variance.
