# Supplementary material for: Delivery of a Muscle-Targeted Adeno-Associated Vector Via Ex Vivo Normothermic Perfusion Is Efficient, Durable, and Safe in a Preclinical Porcine Heart Transplant Model
Source: Transpl Int. 2025 Jun 2;38:13971. doi: 10.3389/ti.2025.13971 (PMC12203020; doi:10.3389/ti.2025.13971)
Supplement: Supplementary file 2 [file DataSheet1.docx]

**Supplemental Methods**

***Recombinant AAV Production***


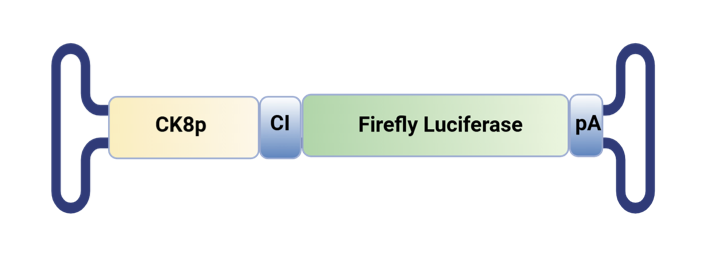


The AAV-SLB101 capsid is based on the AAV9 serotype with modifications for improved transduction efficiency and biodistribution to muscle cells. A single-stranded AAV vector encoding AAV2 ITRs was designed with a CK8 promoter (CK8p) derived from the mouse creatinine kinas gene followed by a chimeric intron driving expression of a firefly luciferase reporter gene. Transcripts are terminated with a small polyadenylation signal sequence. Suspension HEK293 were expanded in F17™ media to reach a volume of 500L and then transfected with Trangene:Rep/Cap:Helper. Cells were chemically lysed and harvested prior to purification.  Downstream purification was then executed using a proprietary platform process for AAV vector clarification, affinity purification, full enrichment through cesium chloride centrifugation, and final formulation through tangential flow filtration.

***Tissue Lysate Preparation***

Tissue samples (150-300 mg) were mechanically homogenized using a Dounce homogenizer with passive lysis buffer (Promega, Cat. #E1941). The lysate was incubated on ice for 30 minutes before being centrifuged at 12,000 g for 10 minutes at 4°C. The protein concentration of the resulting supernatant was determined using the Pierce BCA Protein Assay Kit (Thermo Fisher, Cat. #23225) and a biokinetics reader (EL-340; BioTek Instruments, Winooski, VT). Equivalent protein amounts (400 µg) of the supernatant were used in the luciferase activity assay.

***Luciferase enzymatic activity assay***

All luminometry was performed using a Veritas luminometer (Turner Biosystems, Sunnyvale, CA). Luciferase assay reagent (Promega, Cat. #E1483) was added to each well (50 µL), following the manufacturer's protocol, which included a 2-second measurement delay followed by a 10-second measurement read. Light emission per well was measured in relative light units (RLU). All samples from a single recipient pig were analyzed on individual plates. To minimize the effect of reagent’s chemical decay-related variation, an additional assay was performed with a representative sample from each of the four recipients on the same 96-well plate to normalize the final values.

***Histology and immunofluorescent staining***

Tissue samples from the allograft, native heart, and extracardiac organs were prepared by embedding in Optimal Cutting Temperature (OCT) compound for cryosectioning or flash frozen in liquid nitrogen for molecular analysis. Frozen OCT-embedded tissues were sectioned at 10µm thickness and stained with Hematoxylin and Eosin to evaluate structural changes. For the detection of luciferase expression, OCT-embedded tissues were sectioned at 10µm thickness and stained using a two-step immunofluorescence protocol. This involved the application of a primary mouse anti-luciferase monoclonal antibody (Sigma-Aldrich, Catalog no. L2164, St. Louis, MO) at a dilution of 1:150, followed by a goat anti-mouse secondary antibody conjugated to Alexa Fluor 594 (Abcam, Catalog no. ab150116, Cambridge, UK) diluted 1:300. Imaging of stained sections was performed with a Zeiss 780 upright confocal microscope (Carl Zeiss Microscopy, White Plains, NY). Representative sections of heart and organ tissue were stained with hematoxylin and eosin. These slides were evaluated by a trained cardiovascular pathologist for any signs of myocarditis, inflammation, fibrosis, or edema.

***DNA Isolation***

DNA was isolated from various tissue sections and OCS samples using a KingFisher Apex robotic platform (ThermoFisher, Cat# 5400920). The tissues processed were derived from various heart regions (LA, SA, RA, LV, IVS, RV, apex), psoas muscle, aorta, liver, and lung. Briefly, approximately 30 mg of tissue was homogenized in a homogenization solution (Promega, Cat# AX7890). Then, 200 µL of the homogenized tissue was transferred to a 96 deep well plate format, followed by the addition of lysis and Proteinase K digest solution (Promega, Cat# AX7890).

***Vector Copy Number (VCN) Assay***

A vector copy number assay was developed and carried out to assess the vector copy numbers using primer/probes to the CK8-Luciferase genomes in the isolated tissues. The assay was performed for DNA isolated from the tissues via digital droplet polymerase chain reaction (ddPCR) using a primer/probe set specific for the luciferase gene: forward primer, 5’-GTGGTGTGCAGCGAGAATAG-3’; reverse primer, 5’-CGCTCGTTGTAGATGTCGTTAG-3’; probe, 5’-FAM/TTGCAGTTCTTCATGCCCGTGTTG/TAMSp-3’ . DNA isolated from the tissue samples was diluted to 1 ng/µL using the ddPCR sample dilution solution, and 10 µL was used in each reaction. The ddPCR reaction was carried out using established methods[16]. Briefly, a master mix was prepared using the ddPCR Supermix for Probes (No dUTP) from Bio-Rad (Cat# 1863023), ultrapure water, and a luciferase gene-specific primer-probe mix. serial dilutions were accounted for in the illustration of each graph.

**Anti-Vector Antibody Assessment**

96-well Immulon™ high binding plates were coated with Pig IgG standard. The pig IgG standard was prepared by doing a total of sixteen two-fold dilutions starting at 5ug of protein. 100uL of each standard was added to columns 1-4 of the plate in duplicates. 100ul of AAV-SLB101 at a concentration of 1.75 E+11 capsids/mL were coated on the rest of the wells. The assay plate was sealed and incubated overnight at 2-8°C. The following day, the assay plate was washed three times with 150uL of wash buffer (1x PBS + 0.05% Tween) and blocked with 150uL of 1x blocker casein (ThermoFisher, Cat: 37582) in all wells for 1hr on a plate shaker at 400rpm. On a separate 96-well plate, pig plasma samples were diluted 1:2 using the blocker casein and incubated for 30 mins at RT on plate shaker at 400rpm. The assay plate was then washed x3 with 150uL of wash buffer. After washing, 100uL of samples were added to the assay plate and 100ul of blocker casein to standard wells. The samples were then incubated for 1.5hrs on a plate shaker at 400rpm. Wash steps were repeated as directed. The assay plate was then probed using 100 ul of enzyme conjugated detection antibody for an hour on a plate shaker at 400 rpm. The wash steps were repeated as directed above and then 100uL of 3,3′, 5,5′ tetramethylbenzidine dihydrochloride substrate solution was added per well and incubated in dark for 20 minutes. This reaction was stopped using 100uL of 0.16M sulfuric acid stop solution (ThermoFisher Cat: N600) to each well. The absorbance was read at 450 nm using the Spectramax i3x™ instrument (Molecular Devices, San Jose, CA).

**Supplemental Figures and Tables**


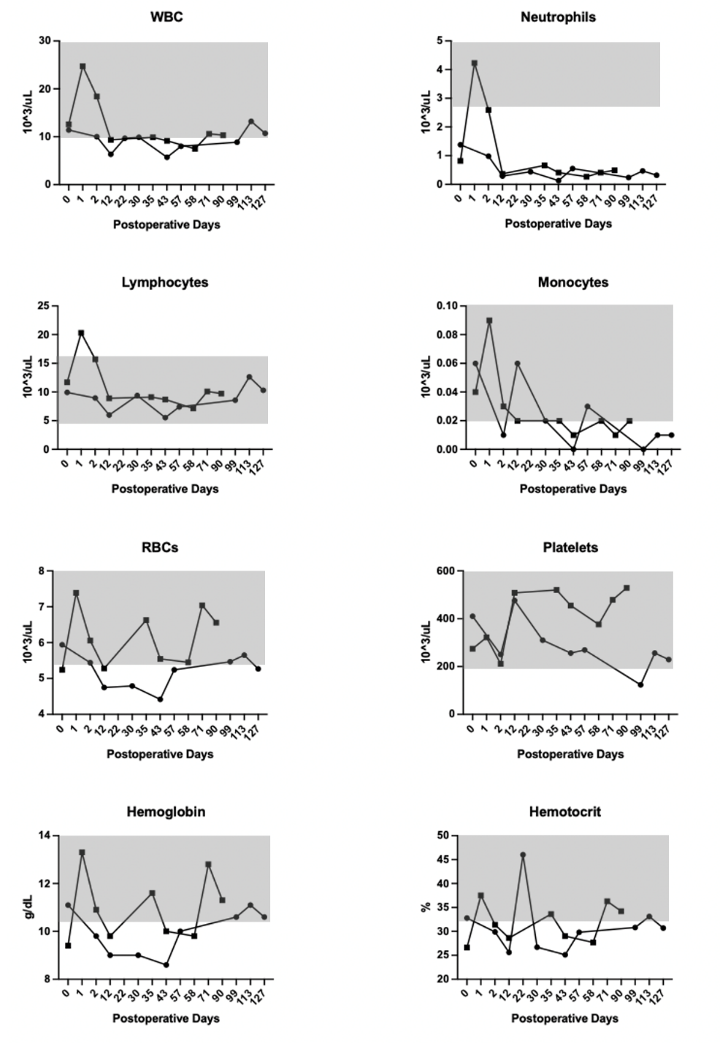


**Supplemental Figure 1: Hematologic laboratory results for recipients *that were survived to 90 and 120 days.*** *Normal laboratory ranges are displayed as gray regions. WBC, white blood count (10.20-30 103/uL); Neutrophils (2.8-16.10 103/uL); Lymphocytes (4.8-16.20 103/uL); Monocytes (0.20-2.25 103/uL); RBCs, red blood cells (5.5-9 106/uL); Platelets (200-1000 103/uL); Hemoglobin (10-16 g/dl); Hematocrit (33-52%)*

**Supplemental Figure 2: Chemistry and electrolyte laboratory results.** *Electrolyte and chemistry laboratory values for both recipients that were survived to 90 and 120 days. Normal ranges are displayed as gray regions. Sodium (131-151 mEq/l); Potassium (3.7-6.1 mEq/l); Chloride (93-108 mEq/l); Glucose (75-136 mg/dl); BUN, blood urea nitrogen (4.0-18.0 mg/dL); Creatinine (0.5-1.1 mg/dL); Calcium (9.9-12.5 mg/dl); Phosphorus (6.3-11.5 mg/dl)*


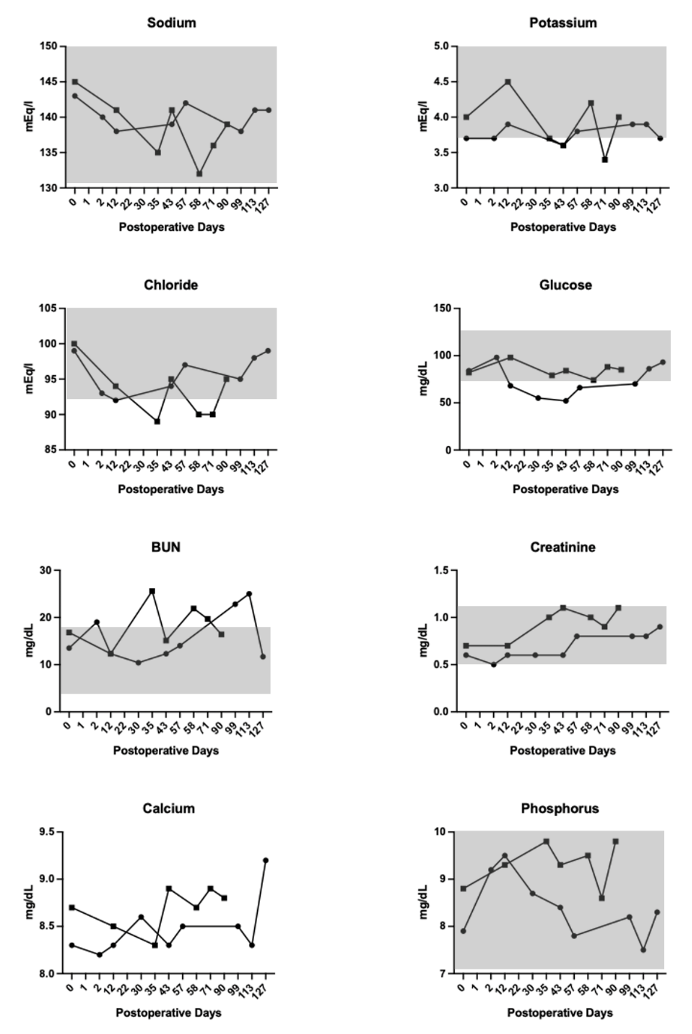


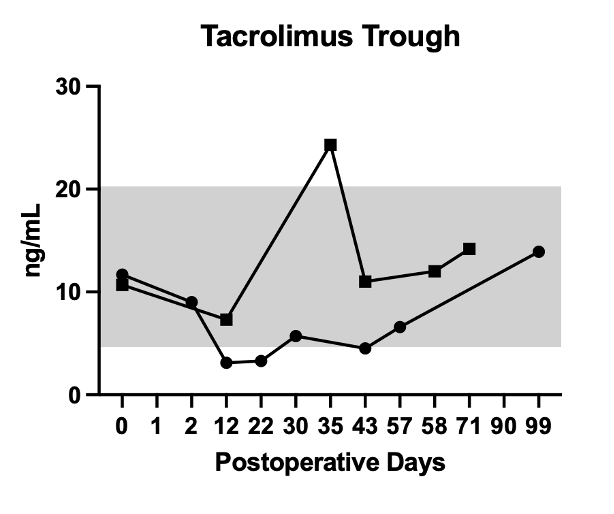

**Supplemental Figure 3: Tacrolimus trough levels for both recipients that were survived to 90 and 120 days.** *Therapeutic window is displayed as a gray region. Therapeutic window (5-20 ng/mL)*

**
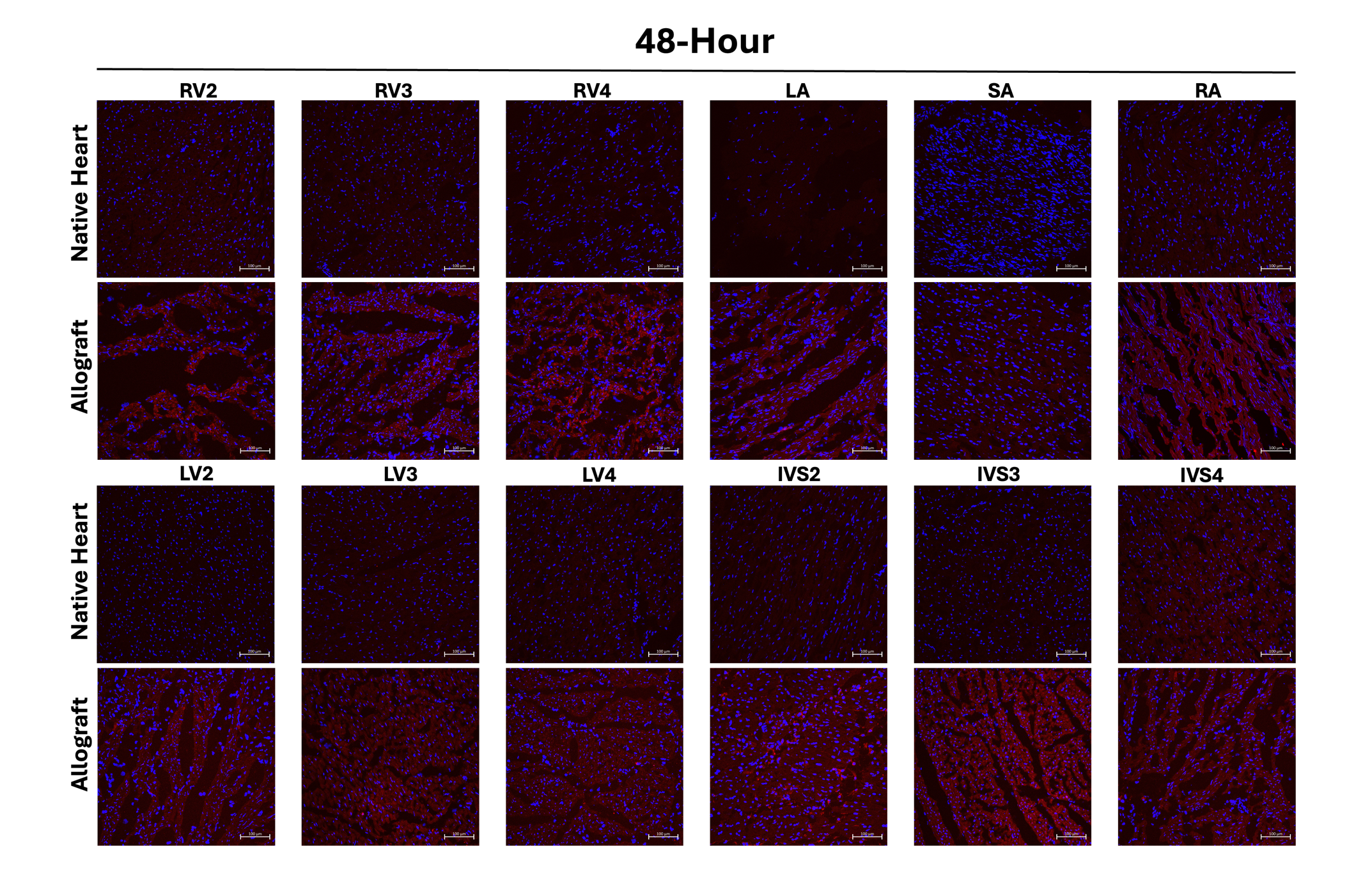
**

**Supplemental Figure 4. Myocardial luciferase expression at 48 hours postoperatively.** *Immunofluorescence staining for luciferase protein expression (red) in the recipient allograft treated with 1x10^14^ VGC AAV-SLB101-Luc. All images are at 20X magnification, at the same exposure time with the scale bar indicating 100 μm. LA, left atrium; SA, atrial septum; RA, right atrium; LV, left ventricle; RV, right ventricle; IVS, interventricular septum.*

*
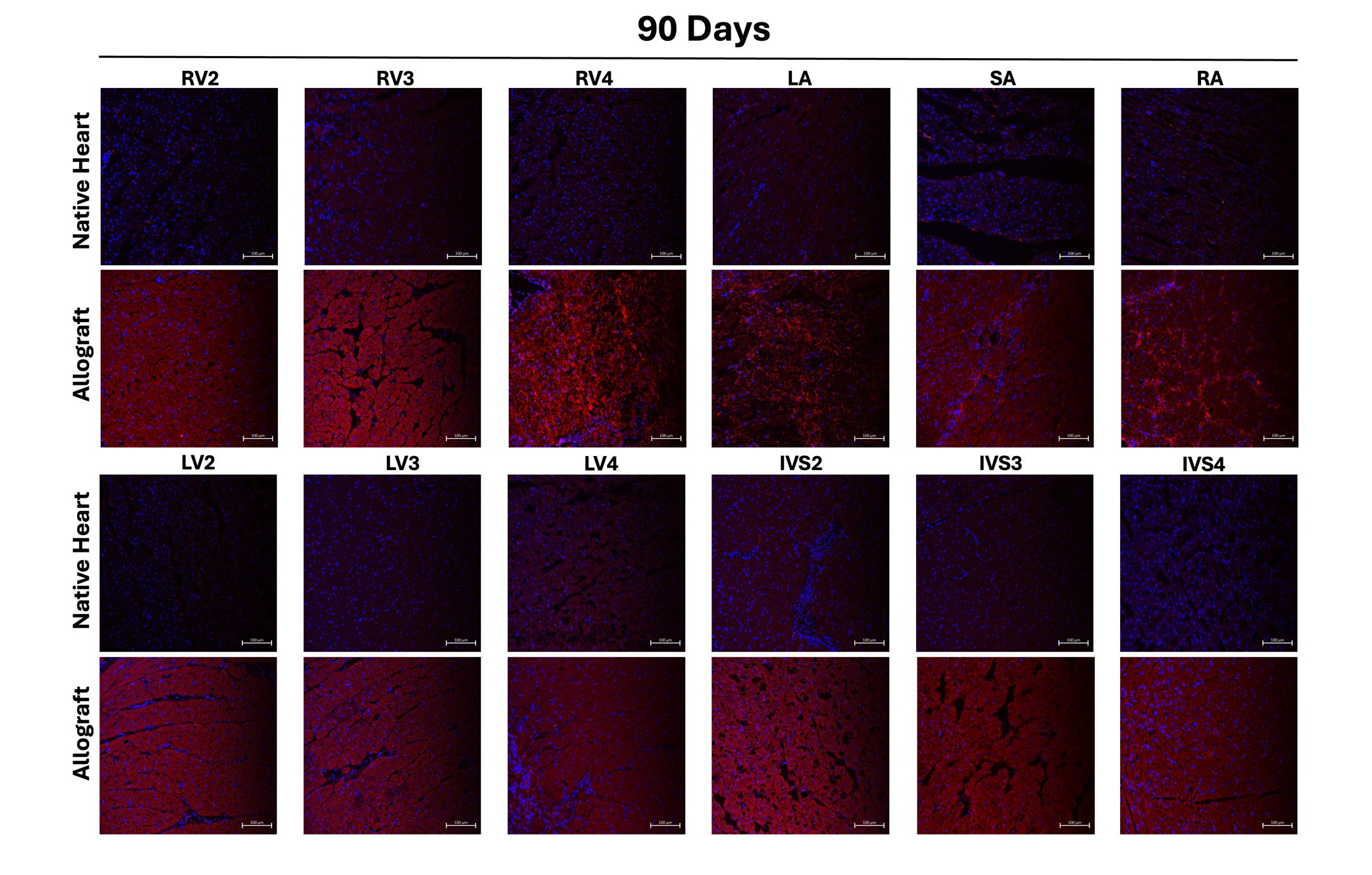
*

**Supplemental Figure 5: Myocardial luciferase expression at 90 days postoperatively.**  *Luciferase expression in the recipient allograft treated with 1x10^14^ VGC AAV-SLB101-Luc and native untreated heart were assessed using immunofluorescent staining (red). All images are at 20X magnification at the same exposure time, with the scale bar indicating 100 μm. LA, left atrium; SA, atrial septum; RA, right atrium; LV, left ventricle; RV, right ventricle; IVS, interventricular septum.*


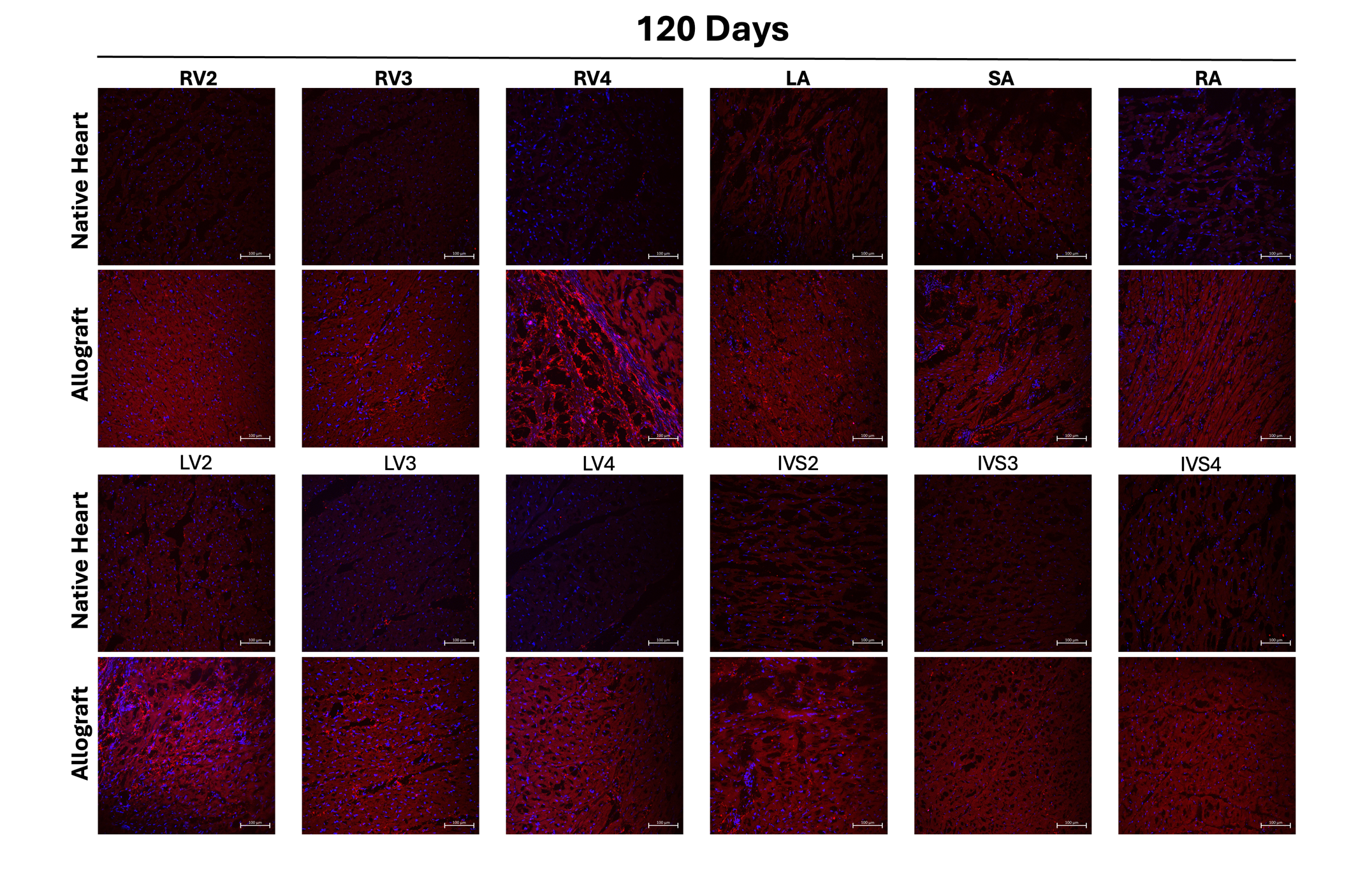


**Supplemental Figure 6: Myocardial luciferase expression at 120 days postoperatively.**  *Luciferase expression in the recipient allograft treated with 1x10^14^ VGC AAV-SLB101-Luc and native untreated heart were assessed using immunofluorescent staining (red). All images are at 20X magnification at the same exposure time, with the scale bar indicating 100 μm. LA, left atrium; SA, atrial septum; RA, right atrium; LV, left ventricle; RV, right ventricle; IVS, interventricular septum.*


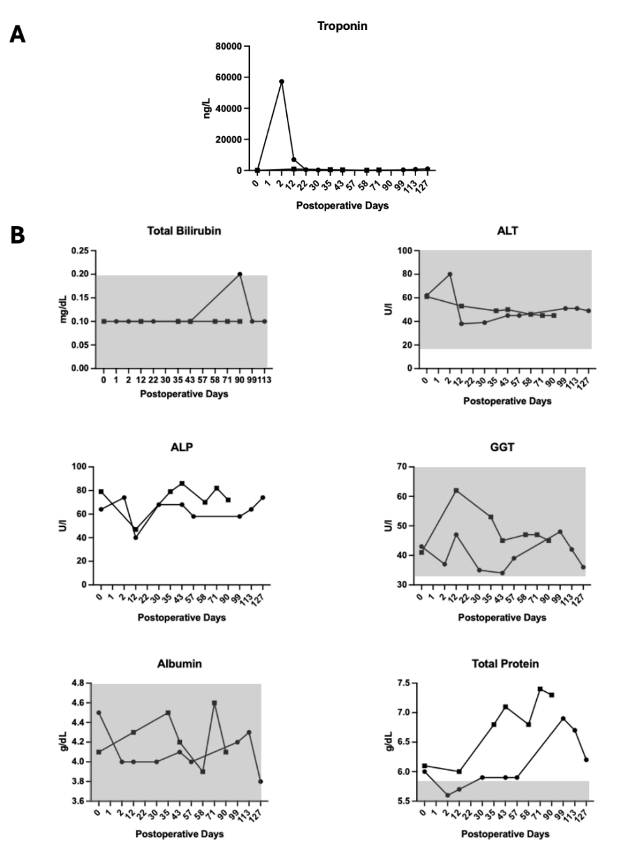
**Supplemental Figure 7: Troponin and liver function laboratory results.** *Serum troponin levels remained within normal limits outside of the immediate post-operative period throughout the duration of follow-up for the two longest-living recipients. Liver laboratory values remained within normal limits for both recipients that were survived to 90 and 120 days. Normal ranges are displayed as gray regions. Troponin (3-70 ng/L); Tbili, total bilirubin (0.0-0.2 mg/dL); ALT, alanine aminotransferase (17-108 U/L); ALP, alkaline phosphatase (130-513 U/L); GGT, gamma-glutamyl transferase (33-94 U/L); Albumin (3.1-4.8 g/dl); total protein (4-5.8 g/dl)*


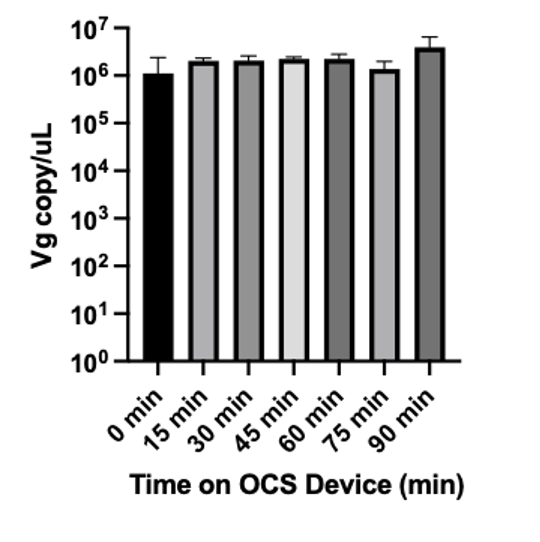


**Supplemental Figure 8: Viral DNA within the OCS perfusate over time.** *Approximately 98% of viral DNA was taken up within the first 15 minutes. Viral DNA remaining within the perfusate did not largely change throughout the remainder of the 2 hour ex-vivo perfusion*


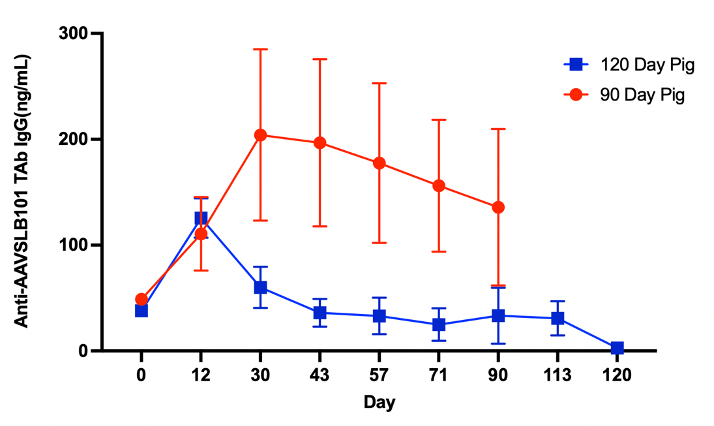


**Supplemental Figure 9: Anti-AAV-SLB101 capsid Ab in recipients over time.** *ELISA was used to measure titers of anti-AAV-SLB101 capsid antibodies present in the plasma of recipients. Anti-AAV-SLB101 capsid antibodies peaked early after transplant and declined over time.*

**Supplemental Table 1.** *Characterization of Lab, Drug, and Timing Parameters during 2-Hour Ex-Vivo Perfusion*

See excel spreadsheet provided

**Supplemental Table 2.** *Luminescence Intensity of Different Sampling Points in Allograft Using Luciferase Assay (RLU per 1µg Homogenized Cardiac Protein).*

| Sample Time Point | | 24H (RLU) | 48H (RLU) | 90D (RLU) | 120D (RLU) |
| --- | --- | --- | --- | --- | --- |
| Atrium | LA | 0 | 3 | 267 | 607 |
|  | SA | 0 | 109 | 550 | 271 |
|  | RA | 0 | 100 | 490 | 1654 |
| Ventricle | LV2a | 0 | 9 | 673 | 3457 |
|  | LV3a | 0 | 5 | 381 | 10129 |
|  | LV4a | 0 | 19 | 1006 | 1751 |
|  | IVS2a | 0 | 13 | 804 | 6808 |
|  | IVS3a | 0 | 21 | 489 | 1031 |
|  | IVS4a | 0 | 17 | 552 | 8439 |
|  | RV2a | 0 | 55 | 3306 | 2701 |
|  | RV3a | 0 | 115 | 666 | 1939 |
|  | RV4a | 0 | 271 | 1338 | 2996 |
|  | LV2p | 0 | 29 | 986 | 7071 |
|  | LV3p | 0 | 179 | 3267 | 6373 |
|  | LV4p | 0 | 336 | 485 | 2134 |
|  | IVS2p | 0 | 12 | 919 | 15201 |
|  | IVS3p | 0 | 33 | 1073 | 6732 |
|  | IVS4p | 0 | 15 | 386 | 2705 |
|  | RV2p | 0 | 42 | 294 | 3385 |
|  | RV3p | 0 | 17 | 2211 | 1739 |
|  | RV4p | 0 | 25 | 655 | 6986 |
|  | LV2m | 0 | 95 | 596 | 2954 |
|  | LV3m | 0 | 13 | 5784 | 1509 |
|  | LV4m | 0 | 128 | 587 | 1743 |
|  | IVS2m | 0 | 3 | 5693 | 2596 |
|  | IVS3m | 0 | 46 | 856 | 6984 |
|  | IVS4m | 0 | 21 | 620 | 10816 |
|  | RV2m | 0 | 15 | 639 | 6515 |
|  | RV3m | 0 | 17 | 2415 | 885 |
|  | RV4m | 0 | 69 | 739 | 2625 |
|  | Apex | 0 | 171 | 1489 | 17379 |

**Supplemental Table 3.** *Luminescence Intensity at Different Timepoints in Recipient’s Native Heart and Extracardiac Organs Using Luciferase Assay (RLU per 1µg Homogenized Tissue Protein)*

| Sample Time Point | | 24H (RLU) | 48H (RLU) | 90D (RLU) | 120D (RLU) |
| --- | --- | --- | --- | --- | --- |
| Atrium | LA | 0 | 1 | 0 | 10 |
|  | SA | 0 | 0 | 0 | 1 |
|  | RA | 0 | 0 | 0 | 1 |
| Ventricle | LV2a | 0 | 0 | 0 | 13 |
|  | LV3a | 0 | 0 | 0 | 3 |
|  | LV4a | 0 | 0 | 0 | 3 |
|  | IVS2a | 0 | 0 | 0 | 6 |
|  | IVS3a | 0 | 0 | 0 | 4 |
|  | IVS4a | 0 | 0 | 0 | 0 |
|  | RV2a | 0 | 0 | 0 | 1 |
|  | RV3a | 0 | 0 | 0 | 6 |
|  | RV4a | 0 | 0 | 0 | 2 |
|  | LV2p | 0 | 0 | 0 | 0 |
|  | LV3p | 0 | 0 | 0 | 0 |
|  | LV4p | 0 | 0 | 0 | 0 |
|  | IVS2p | 0 | 0 | 0 | 0 |
|  | IVS3p | 0 | 0 | 0 | 1 |
|  | IVS4p | 0 | 0 | 0 | 0 |
|  | RV2p | 0 | 0 | 0 | 0 |
|  | RV3p | 0 | 1 | 0 | 0 |
|  | RV4p | 0 | 0 | 0 | 0 |
|  | LV2m | 0 | 1 | 0 | 0 |
|  | LV3m | 0 | 0 | 0 | 0 |
|  | LV4m | 0 | 0 | 0 | 0 |
|  | IVS2m | 0 | 0 | 0 | 0 |
|  | IVS3m | 0 | 0 | 0 | 0 |
|  | IVS4m | 0 | 0 | 0 | 0 |
|  | RV2m | 0 | 0 | 0 | 0 |
|  | RV3m | 0 | 0 | 0 | 0 |
|  | RV4m | 0 | 0 | 0 | 0 |
|  | Apex | 0 | 0 | 0 | 34 |
| Extra-cardiac | Lung | 0 | 0 | 0 | 0 |
|  | Liver | 0 | 0 | 0 | 0 |
|  | kidney | 0 | 0 | 0 | 0 |
|  | Psoas m | 0 | 0 | 0 | 0 |

**Supplemental Table 4: Pathological grading of recipient allografts (treated with 1x10^14^ VGC AAV-SLB101-Luc), native heart, liver, psoas muscle and lung tissue.** *An expert blinded cardiac pathologist reviewed representative H&E stains of each chamber of both the treated allograft and native heart for evidence of rejection, myocarditis, inflammation, or edema. Each sample was graded for rejection on the validated International Society of Heart and Lung Transplant scale (0R, minimal/none; 1R, mild; 2R, moderate; 3R, severe).*

| **24 Hours** | | | | | |
| --- | --- | --- | --- | --- | --- |
| **Native Heart** | | | **Allograft** | | |
| **Sample** | **ISHLT Acute Cellular Rejection Grade** | **Myocarditis, Inflammatory Changes, Edema** | **Sample** | **ISHLT Acute Cellular Rejection Grade** | **Myocarditis, Inflammatory Changes, Edema** |
| RA | 0R | none/minimal | RA | 0R | mild |
| LA | 0R | none/minimal | LA | 0R | none/minimal |
| SA | 0R | none/minimal | SA | 0R | none/minimal |
| LV | 0R | none/minimal | LV | 0R | none/minimal |
| RV | 0R | none/minimal | RV | 0R | none/minimal |
| IVS | 0R | none/minimal | IVS | 0R | none/minimal |
| Psoas muscle | 0R | none/minimal |  |  |  |
| Liver | 0R | none/minimal |  |  |  |
| Lung | 0R | none/minimal |  |  |  |
| **48 Hours** | | | | | |
| **Native Heart** | | | **Allograft** | | |
| **Sample** | **ISHLT Acute Cellular Rejection Grade** | **Myocarditis, Inflammatory Changes, Edema** | **Sample** | **ISHLT Acute Cellular Rejection Grade** | **Myocarditis, Inflammatory Changes, Edema** |
| RA | 0R | none/minimal | RA | 0R | none/minimal |
| LA | 0R | none/minimal | LA | 2R | moderate |
| SA | 0R | none/minimal | SA | 0R | none/minimal |
| LV | 0R | none/minimal | LV | 2R | moderate |
| RV | 0R | none/minimal | RV | 2R | moderate |
| IVS | 0R | none/minimal | IVS | 1R | mild |
| Psoas muscle | 0R | none/minimal |  |  |  |
| Liver | 0R | none/minimal |  |  |  |
| Lung | 0R | none/minimal |  |  |  |
| **90 Days** | | | | | |
| **Native Heart** | | | **Allograft** | | |
| **Sample** | **ISHLT Acute Cellular Rejection Grade** | **Myocarditis, Inflammatory Changes, Edema** | **Sample** | **ISHLT Acute Cellular Rejection Grade** | **Myocarditis, Inflammatory Changes, Edema** |
| RA | 0R | none/minimal | RA | 0R | none/minimal |
| LA | 0R | none/minimal | LA | 0R | none/minimal |
| SA | 0R | none/minimal | SA | NA | NA |
| LV | 0R | none/minimal | LV | 0R | none/minimal |
| RV | 0R | none/minimal | RV | 1R | mild |
| IVS | 0R | none/minimal | IVS | 0R | none/minimal |
| Psoas muscle | 0R | none/minimal | EMB30 | no evaluable myocardial tissue | no evaluable myocardial tissue |
| Liver | 0R | none/minimal | EMB60 | no evaluable myocardial tissue | no evaluable myocardial tissue |
| Lung | 0R | none/minimal |  |  |  |
| **120 Days** | | | | | |
| **Native Heart** | | | **Allograft** | | |
| **Sample** | **ISHLT Acute Cellular Rejection Grade** | **Myocarditis, Inflammatory Changes, Edema** | **Sample** | **ISHLT Acute Cellular Rejection Grade** | **Myocarditis, Inflammatory Changes, Edema** |
| RA | 0R | none/minimal | RA | 0R | none/minimal |
| LA | 0R | none/minimal | LA | 0R | none/minimal |
| SA | 0R | none/minimal | SA | 0R | none/minimal |
| LV | 0R | none/minimal | LV | 0R | none/minimal |
| RV | 0R | none/minimal | RV | 0R | none/minimal |
| IVS | 0R | none/minimal | IVS | 0R | none/minimal |
| Psoas muscle | 0R | none/minimal |  |  |  |
| Liver | 0R | none/minimal |  |  |  |
| Lung | 0R | none/minimal |  |  |  |
